# Supplementary material for: Complexome profiling of the Chlamydomonas psb28 mutant reveals TEF5 as an early PSII assembly factor
Source: Plant Cell. 2025 Mar 17;37(6):koaf055. doi: 10.1093/plcell/koaf055 (PMC12164590; doi:10.1093/plcell/koaf055)
Supplement: koaf055_Supplementary_Data [file koaf055_supplementary_data.zip › psb28_interactive.zip!/plots/intensity/qualityPlots/peptides/SECY1_Cre16.g681900.t1.2.html]

### Cre16.g681900.t1.2

Trivial name: SECY1  
SeqCoverage [median]: 19.50  
Mass: 55.99 kDa  
  
 

| Sequence | MissedCleavage | Mass [Da] | wtIntensity | muIntensity | Score |
| --- | --- | --- | --- | --- | --- |
| AKFQYYQK | 1 | 1074.55 | 361.59 | 583.31 | 116.88 |
| DLAEQLK | 0 | 815.44 | 1121.17 | 4369.32 | 168.45 |
| DLAEQLKK | 1 | 943.53 | 0.00 | 9565.47 | 130.22 |
| FQYYQK | 0 | 875.42 | 0.00 | 940.48 | 143.88 |
| IPVNYSNR | 0 | 961.50 | 4145.47 | 6811.73 | 101.33 |
| LSSGNLAR | 0 | 816.45 | 33351.55 | 43174.32 | 187.34 |
| LYDNMKM | 1 | 913.40 | 0.00 | 481.39 | 120.65 |
| QSYLPFK | 0 | 881.46 | 5023.03 | 11937.75 | 107.67 |
| QTAEYVTR | 0 | 966.48 | 12473.78 | 10439.50 | 93.37 |
| RIPVNYSNR | 1 | 1117.60 | 5498.29 | 6341.99 | 105.36 |
| TGAAIPAIRPGK | 1 | 1150.68 | 29103.07 | 43214.65 | 106.62 |
| TGAAIPAIRPGKQTAEYVTR | 2 | 2099.15 | 586.44 | 929.56 | 58.02 |
| VGVYIR | 0 | 705.42 | 0.00 | 3217.87 | 130.16 |
| YQDVDK | 0 | 766.35 | 813.32 | 634.27 | 100.19 |
| YQDVDKLYDNMKM | 2 | 1661.74 | 9575.47 | 13793.00 | 107.60 |
|  |  |  | 102053.2 | 156434.6 |  |
